# Procalcitonin decrease over 72 hours in US critical care units predicts fatal outcome in sepsis patients

**DOI:** 10.1186/cc12787

**Published:** 2013-06-20

**Authors:** Philipp Schuetz, Paula Maurer, Vikas Punjabi, Ami Desai, Devendra N Amin, Eric Gluck

**Affiliations:** 1 Harvard School of Public Health, 677 Huntington Avenue, Boston, MA 02115, USA; 2 Medical/Surgical Intensive Care Unit, Morton Plant Hospital, 300 Pinellas Street, Clearwater, FL 33756, USA; 3 Department of Critical Care Medicine, Swedish Covenant Hospital, 5145 N. California Ave, Chicago, IL 60625-3642, USA

**Keywords:** Sepsis, Severe sepsis, Septic shock, Biomarker, Procalcitonin, Procalcitonin kinetics, Procalcitonin change, Mortality, Mortality prediction, APACHE IV, SAPS II, Clinical risk score

## Abstract

**Introduction:**

Close monitoring and repeated risk assessment of sepsis patients in the intensive care unit (ICU) is important for decisions regarding care intensification or early discharge to the ward. We studied whether considering plasma kinetics of procalcitonin, a biomarker of systemic bacterial infection, over the first 72 critical care hours improved mortality prognostication of septic patients from two US settings.

**Methods:**

This retrospective analysis included consecutively treated eligible adults with a diagnosis of sepsis from critical care units in two independent institutions in Clearwater, FL and Chicago, IL. Cohorts were used for derivation or validation to study the association between procalcitonin change over the first 72 critical care hours and mortality.

**Results:**

ICU/in-hospital mortality rates were 29.2%/31.8% in the derivation cohort (*n *= 154) and 17.6%/29.4% in the validation cohort (*n *= 102). In logistic regression analysis of both cohorts, procalcitonin change was strongly associated with ICU and in-hospital mortality independent of clinical risk scores (Acute Physiology, Age and Chronic Health Evaluation IV or Simplified Acute Physiology Score II), with area under the curve (AUC) from 0.67 to 0.71. When procalcitonin decreased by at least 80%, the negative predictive value for ICU/in-hospital mortality was 90%/90% in the derivation cohort, and 91%/79% in the validation cohort. When procalcitonin showed no decrease or increased, the respective positive predictive values were 48%/48% and 36%/52%.

**Discussion:**

In septic patients, procalcitonin kinetics over the first 72 critical care hours provide prognostic information beyond that available from clinical risk scores. If these observations are confirmed, procalcitonin monitoring may assist physician decision-making regarding care intensification or early transfer from the ICU to the floor.

## Introduction

Accurately assessing risk of poor outcome in intensive care unit (ICU) and other critical care patients with sepsis is challenging, but may help improve patient management and decrease sepsis-related morbidity and mortality. Since prompt and specific treatment in those with severe sepsis and septic shock has been shown to improve outcomes [1], it is vital to ensure a timely diagnosis of this disease process. Once stabilized in the critical care setting, sepsis patients may be transferred to a medical ward for further treatment. In clinical practice, appropriate transfer decisions are important, as treatment failure on the floor leading to ICU readmission is associated with adverse prognosis and prolonged stays [2]. However, such decisions are challenging, because the clinical presentation may not always reflect readiness for discharge. Therefore, applying an accurate risk stratification tool after prompt diagnosis and treatment of sepsis in critical care units would allow identification of patients at low risk for adverse outcome who are ready for ICU discharge and, at the same time, of patients at high risk who need intensification of therapy.

One candidate risk stratification tool is procalcitonin (PCT), the 116-amino acid prohormone of the calcium metabolism regulator, calcitonin [3]. PCT is a biomarker of systemic bacterial infection and sepsis, because although mature calcitonin is exclusively produced by thyroid gland C-cells, PCT is synthesized in numerous extra-thyroidal tissues in response to lipopolysaccharides and bacterially-induced cytokines [4]. Plasma PCT levels rise within approximately three to six hours of initial clinical manifestation of sepsis and fall if the sepsis is controlled.

Previous research, mainly from Europe, showed that changes over time in biomarkers including PCT and C-reactive protein (CRP) may be used to monitor patients with sepsis, respiratory infections or both in the ICU [5-11]. These studies have suggested that a decrease in PCT concentration may help guide physicians on when to de-escalate antibiotic therapy in medical as well as surgical ICU patients [6-11]. European studies also showed that PCT kinetics have prognostic implications, as falling values correlate with good outcomes, while static or increasing values correlate with adverse outcomes including mortality [12-14]. However, there are as yet no accepted cut-offs for risk assessment in ICU patients, and reports from US settings are largely lacking.

We therefore retrospectively analyzed data from patients in critical care units with a diagnosis of sepsis (confirmed or probable) from two independent US health care institutions to investigate the prognostic information obtainable from the change in PCT concentration from the admission PCT level and a repeated PCT measurement 72 critical care hours later. The analysis sought to derive and preliminarily validate cut-off levels for mortality risk stratification that could be further validated in prospective trials.

## Materials and methods

### Study design, patients and setting

This observational study included two independent cohorts of adults consecutively admitted to critical care units for the main diagnosis of sepsis based on International Classification of Diseases edition 9 coding and medical record review. Upfront, we designated as a derivation cohort the larger group, patients treated at an 18-bed adult medical-surgical ICU, at Morton Plant Hospital, Clearwater, Florida, a 687-bed community referral center, between January 2009 and April 2010. All patients in this cohort fulfilled the criteria of either severe sepsis (18%) or septic shock (82%) as prospectively routinely assessed in the institutional sepsis surveillance protocol. We assigned as a validation cohort the patients hospitalized in a 14-bed adult medical-surgical ICU or a 16-bed adult intermediate care unit at the Swedish Covenant Hospital, Chicago, Illinois, a 350-bed inner-city tertiary referral center, between January 2009 and March 2011. All patients in the Swedish Covenant cohort had a systemic bacterial infection as a main diagnosis and were considered by the treating physicians to have sepsis. However, due to our retrospective study design, it was not possible to confirm that each fulfilled criteria for severe sepsis or septic shock. To be eligible for either cohort, patients needed to have one PCT measurement upon admission (within 24 hours) and one draw 72 (± 12) hours thereafter during an uninterrupted critical care stay. Retrospective collection and analysis of data from each cohort was approved beforehand by the respective local institutional review board (IRB) (that is, Pasco-Pinellas IRB of Morton Plant Mease Health Care, Florida and the Swedish Covenant IRB, Chicago, IL). Both IRBs granted a waiver for informed consent since the study was anonymous and, because of its retrospective nature, entailed no patient interventions.

### Data collection

For the derivation cohort, data were extracted using ICU Tracker® (Alere Informatics Solutions, Charlottesville, VA, USA), an electronic acquisition database drawing from electronic feeds of demographic, clinical, vital signs, laboratory, co-morbidity, complication, pharmacy and billing information. ICU and hospital length-of-stay (LOS), Acute Physiology, Age and Chronic Health Evaluation (APACHE) IV score at ICU admission and observed mortality rates also were obtained through ICU Tracker®. Additional data were acquired using Theradoc® (Hospira, Lake Forest, IL, USA), an electronic infection control surveillance product. Further, ICU charts were manually reviewed for data verification in case of uncertainty.

Similarly, for the validation cohort, we extracted data from all patients' electronic medical records of their hospital stays. In particular, we gathered and used for the analysis: (a) patient identification number; (b) socio-demographic characteristics; (c) laboratory results including all PCT measurements; (d) Simplified Acute Physiology Score (SAPS) II at critical care unit admission; (e) co-morbidities based on Diagnosis-Related Group codings; (f) sepsis-specific medication(s); (g) ICU and hospital LOS; and (h) vital status during the ICU and hospital stay.

### PCT measurement

For PCT measurement, the Clearwater institution used the VIDAS B.R.A.H.M.S PCT assay (bioMérieux, Marcy L'Etoile, France), an automated heterogeneous sandwich immunoassay with fluorescence detection. Total assay time of the VIDAS method is 20 minutes with a measuring range of 0.05 to 200 μg/L and a functional sensitivity of 0.09 μg/L. The Chicago institution used a time-resolved amplified cryptate emission technology assay (B·R·A·H·M·S PCT sensitive KRYPTOR®, Thermo Scientific Biomarkers, Hennigsdorf, Germany) with a 0.019 µg/L lower limit of detection, a 0.06 μg/L functional sensitivity, and ≤3% intra-assay or inter-assay coefficients of variation [15]. Both methods have shown an excellent correlation and concordance and both can be used with the same nominal PCT cut-off ranges in clinical routine [16]. The initial PCT result in all patients was on the day of ICU admission. In patients in whom more than one follow-up PCT measurement was available for the period 72 ± 12 hours after the initial value, the one closest to 72 hours was used for the study analyses.

### Hypothesis and statistics

We hypothesized that the magnitude of the relative change in PCT concentration within 24 hours of ICU admission until after 72 ± 12 hours in the critical care setting would be associated with all-cause ICU and in-hospital mortality, our primary endpoints. As previously suggested [17], to test this hypothesis, we performed logistic regression analyses and calculated odds ratios (ORs) and their 95% confidence intervals (CIs). To improve the model fit, we transformed relative PCT change into deciles before modeling. In a separate analysis, we also adjusted the models for illness severity using the initial severity of illness score that was routinely calculated in each institution. Specifically, the APACHE IV was used in the derivation cohort and SAPS II in the validation cohort. We assessed the discrimination provided by 72-hour PCT change as a mortality predictor by calculating the area under the receiver operating characteristic curve (AUC of the ROC curve), which reflects the probability that a tested predictive variable/method will correctly categorize an individual. The AUC is derived by plotting the sensitivity (true-positive rate) against 1-the specificity (false-positive rate) associated with given values of the predictive variable/method. An AUC of 0.5 implies that the tested variable/method is no more accurate than a coin toss, whereas an AUC of 1.0 means that the variable/method is always correct; values around 0.7 or greater are generally deemed to suggest that the tested variable/method may have clinical utility. Calibration of the regression models was assessed with Hosmer-Lemeshow Goodness of Fit tests. Additionally, we examined whether combining the PCT change with clinical risk scores, namely APACHE IV (derivation cohort) or SAPS II (validation cohort), at ICU admission, improved mortality prediction accuracy over that attained with the respective score alone. To do so, we compared AUCs of a logistic regression model of the clinical score by itself versus the AUCs of a model combining the clinical score plus the PCT change. We also dichotomized the 72-hour PCT change at four different cut-off levels, namely: (1) 0% decrease or PCT increase; (2) 40% decrease; (3) 60% decrease; and (4) 80% or more decrease. For these cut-offs, we calculated sensitivity, specificity, and positive and negative predictive values.

All statistical analyses were done with SAS 6.12 (SAS Institute, Cary, NC, USA) or STATA 9.2 (Stata Corp., College Station, TX, USA). Mean values are reported with their standard deviation (SD), and median values with their interquartile range (IQR, 25^th ^to 75^th ^percentile). All testing was two-tailed and *P*-values under 0.05 were considered statistically significant.

## Results

### Study cohort characteristics

Characteristics of the two patient populations are presented in Table 1. The derivation and validation cohorts had virtually the same mean age. However, there was a trend towards a statistically significant difference between the groups in gender composition and there were statistically significant differences in racial composition and in distribution of the primary locus for sepsis. Specifically, the derivation cohort was more female and much more Caucasian than was the validation cohort. Additionally, the derivation cohort far more often had the urinary tract and the validation cohort far more often had other sites as the primary locus for sepsis. In both groups, however, the respiratory tract and abdomen were the two commonest primary loci for sepsis, and primary bacteremia with uncertain clinical focus had very similar prevalence.

**Table 1:** Characteristics of the two study cohorts

Variable	Derivation cohort (number = 154)	Validation cohort (number = 102)	*P*
Demographics			
Age (years), mean ± SD	65.8 ± 16.2	65.8 ±17.0	0.97
Female gender, % (number)	55.2% (85)	43.1% (44)	0.06
Race, % (number)			<0.01
White	89.6%(138)	48.0% (49)	
African-American	6.5% (10)	13.7% (14)	
Hispanic	0 0% (0)	15.7% (16)	
Asian	3.9% (6)	12.7% (13)	
Other	0% (0)	9.8% (10)	
Primary locus for sepsis, % (number)			<0.01
Respiratory tract	45.5% (70)	39.2% (40)	
Intra-abdominal	21.4% (33)	13.7% (14)	
Skin	0 (0)	2.0% (2)	
Urinary tract	16.9% (26)	3.9% (4)	
Bacteremia without organ specified	13.0% (20)	13.7% (14)	
Other infections including nervous system, prosthesis, heart, abscesses	3.3% (5)	27.5% (28)	
Type of admission, % (number)			0.68
Medical	83.1% (128)	84.3% (86)	
Surgical	17.6% (26)	15.7% (16)	
Admission risk score, mean ± SD			
APACHE IV	81.8 ± 25.9	NA	
SAPS II	NA	45.3 ± 14.3	
PCT concentrations (µg/L), mean ± SD			
Admission	32.6 ± 40.6	27.4 ± 44.9	0.34
After 72 ± 12 hours in ICU	13.3 ± 21.8	16.1 ± 40.4	0.46
Length-of-stay, days, mean ± SD			
ICU	7.7 ± 6.3	6.9 ± 5.1	0.04
Total hospital	15.9 ± 10.7	15.4 ±10.1	0.66
Mortality, % (number), 95% CI			
ICU	29.2% (45) 22.0% to 36.4%]	17.6% (18) 10.1% to 25.2%	0.04
Total in-hospital	31.8% (49) 24.4% to 39.3%	29.4% (30) 20.4% to 38.5%)	0.68

APACHE, Acute Physiology, Age and Chronic Health Evaluation; CI, confidence interval; ICU, intensive care unit; PCT, procalcitonin; SAPS, Simplified Acute Physiology Score

Mean PCT levels on admission were 17.9% higher in the derivation cohort and, on a relative basis, fell substantially more sharply over the studied interval in that group compared to the validation cohort (61.9% versus 40.9% mean relative decrease). Hospital LOS was comparable between the cohorts, as was in-hospital mortality. However, critical care unit LOS was slightly but significantly longer and ICU mortality was appreciably and significantly higher in the derivation cohort.

### Derivation cohort

In the derivation cohort, the mean (SD) relative individual change in PCT concentration from admission to 72 hours was -46% (± 39%) and the median (IQR) individual change was -66% (-38% to -81%). PCT kinetics over 72 hours were strongly associated with ICU and in-hospital mortality (adjusted OR [95%CI] per 10% PCT increase 1.3 [1.1 to 1.5], *P *= 0.001 and 1.3 [1.1 to 1.4], *P *= 0.001, respectively) (Table 2). The association of the 72-hour PCT change with outcomes (ICU mortality and in-hospital mortality) was assessed using logistic regression analysis. Goodness of Fit testing showed no evidence of miscalibration (*P *= 0.95 and *P *= 0.93). The 72-hour PCT change or the APACHE IV score at ICU admission provided similar discrimination of ICU survivors versus non-survivors (AUC 0.70 versus 0.66) and hospital survivors versus non-survivors (AUC 0.70 versus 0.68). Adding the PCT change to the APACHE IV score in a joint logistic model tended to improve discrimination relative to use of the risk score alone (AUC 0.73, *P *= 0.01 for ICU mortality and AUC 0.75, *P *= 0.06 for in-hospital mortality).

**Table 2:** Association of 72-hour PCT kinetics and ICU and total in-hospital mortality

	Derivation cohort (number = 154)	Validation cohort (number = 102)
Discrimination: AUC (95%CI)		
ICU mortality	0.70 (0.61, 0.79)	0.71 (0.57, 0.84)
In-hospital mortality	0.70 (0.61, 0.78)	0.67 (0.54, 0.79)
Adjusted regression analyses^a ^OR (95%CI), *P *value		
ICU mortality		
Per 10% PCT decrease	1.3 (1.1, 1.5), 0.001	1.3 (1.1, 1.6), 0.01
In-hospital mortality		
Per 10% PCT decrease	1.3 (1.1, 1.4*)*, 0.001	1.2 (1.04, 1.4), 0.012

**^a^**Adjusted for severity using the clinical risk score routinely obtained at ICU admission (APACHE IV score in the derivation cohort, SAPS II score in the validation cohort). AUC, area under the receiver operating characteristic curve; CI, confidence interval; ICU, intensive care unit; OR, odds ratio; PCT, procalcitonin.

Based on ROC analysis, we defined four different 72-hour PCT change cut-offs to separate hospital survivors from non-survivors. The mortality rates within the resultant ranges are presented in Table 3. In the derivation cohort, ICU mortality rose from 9.5% in patients with a PCT decrease of >80% to 47.8% in patients with no decrease or an increase. Results for in-hospital mortality were similar. Table 4 presents sensitivities, specificities, and negative and positive predictive values at the different cut-offs. In the derivation cohort, a PCT decrease of >80% had a high negative predictive value of 90% for ICU mortality with a sensitivity of 91%, while the positive predictive value when PCT did not decrease or increased was 48%, with a specificity of 89%. Results for in-hospital mortality were similar.

**Table 3:** 72-hour PCT kinetics and mortality

PCT kinetics over 72 ± 12 hours	Derivation cohort (number = 154)	Validation cohort (number = 102)
ICU mortality		
PCT increase	47.8% (number = 11/23)	36.0% (number = 9/25)
PCT decrease 0% to 40%	52.9% (number = 9/17)	21.4% (number = 3/14)
PCT decrease 40% to 60%	30.4% (number = 7/23)	25.0% (number = 2/8)
PCT decrease 60% to 80%	28.6% (number = 14/49)	4.8% (number = 1/21)
PCT decrease >80%	9.5% (number = 4/42)	8.8% (number = 3/34)
In-hospital mortality		
PCT increase	47.8% (number = 11/23)	52.0% (number = 13/25)
PCT decrease 0% to 40%	52.9% (number = 9/17)	42.9% (number = 6/14)
PCT decrease 40% to 60%	39.1% (number = 9/23)	25.0% (number = 2/8)
PCT decrease 60% to 80%	32.7% (number = 16/49)	9.5% (number = 2/21)
PCT decrease >80%	9.5% (number = 4/42)	20.6% (number = 7/34)

PCT, procalcitonin

**Table 4:** Sensitivity, specificity, positive and negative predictive values for ICU and in-hospital mortality at different PCT kinetics cut-offs

Cut-off	Sensitivity (95%CI)	Specificity (95%CI)	PPV (95%CI)	NPV (95%CI)
**Part A: ICU mortality**				

	Derivation cohort			
0% PCT decrease	0.24 (0.13-0.40)	0.89 (0.82-0.94)	0.48 (0.27-0.69)	0.74 (0.66-0.81)
40% PCT decrease	0.44 (0.30-0.60)	0.82 (0.73-0.88)	0.50 (0.34-0.66)	0.78 (0.69-0.85)
60% PCT decrease	0.60 (0.44-0.74)	0.67 (0.57-0.76)	0.43 (0.30-0.56)	0.80 (0.71-0.88)
80% PCT decrease	0.91 (0.79-0.98)	0.35 (0.26-0.45)	0.37 (0.28-0.46)	0.90 (0.77-0.97)
	Validation cohort			
0% PCT decrease	0.50 (0.26-0.74)	0.81 (0.71-0.89)	0.36 (0.18-0.57)	0.88 (0.79-0.95)
40% PCT decrease	0.67 (0.41-0.87)	0.68 (0.57-0.78)	0.31 (0.17-0.48)	0.90 (0.80-0.96)
60% PCT decrease	0.78 (0.52-0.94)	0.61 (0.49-0.71)	0.30 (0.17-0.45)	0.93 (0.82-0.98)
80% PCT decrease	0.83 (0.59-0.96)	0.37 (0.27-0.48)	0.22 (0.13-0.34)	0.91 (0.76-0.98)

**Part B: In-hospital mortality**				

	Derivation cohort			
0% PCT decrease	0.22 (0.12-0.37)	0.89 (0.81-0.94)	0.48 (0.27-0.69)	0.71 (0.62-0.79)
40% PCT decrease	0.41 (0.27-0.56)	0.81 (0.72-0.88)	0.50 (0.34-0.66)	0.75 (0.66-0.82)
60% PCT decrease	0.59 (0.44-0.73)	0.68 (0.58-0.76)	0.46 (0.33-0.59)	0.78 (0.68-0.86)
80% PCT decrease	0.92 (0.80-0.98)	0.36 (0.27-0.46)	0.40 (0.31-0.50)	0.90 (0.77-0.97)
	Validation cohort			
0% PCT decrease	0.43 (0.25-0.63)	0.83 (0.73-0.91)	0.52 (0.31-0.72)	0.78 (0.67-0.87)
40% PCT decrease	0.63 (0.44-0.80)	0.72 (0.60-0.82)	0.49 (0.32-0.65)	0.83 (0.71-0.91)
60% PCT decrease	0.70 (0.51-0.85)	0.64 (0.52-0.75)	0.45 (0.30-0.60)	0.84 (0.71-0.92)
80% PCT decrease	0.77 (0.58-0.90)	0.38 (0.26-0.50)	0.34 (0.23-0.46)	0.79 (0.62-0.91)

CI, confidence interval; NPV, negative predictive value; PCT, procalcitonin; PPV, positive predictive value

### Validation cohort

In the validation cohort, the mean (SD) relative change in PCT concentration from admission to 72 hours was -26% (± 26%) and the median (IQR) change was -68% (-1% to -83%). As in the derivation cohort, 72-hour PCT kinetics were significantly associated with ICU and in-hospital mortality in an adjusted logistic regression analysis (adjusted OR [95%CI] per 10% PCT decrease, 1.3 [1.1 to 1.6], *P *= 0.01 and 1.2 [1.04 to 1.4], *P *= 0.012, respectively). Discrimination provided by the PCT change was higher than that provided by the SAPS II score for ICU mortality (AUC 0.71 versus 0.57) and in-hospital mortality (AUC 0.67 versus 0.61). In this cohort, too, addition of 72-hour PCT kinetics to the clinical risk score (here SAPS II rather than APACHE IV) at ICU admission increased the prognostic accuracy (AUC 0.70 and 0.68) relative to that attained with the risk score alone. Again, there was no evidence of miscalibration in Goodness of Fit statistics for either outcome (*P *= 0.51 and *P *= 0.19).

Diagnostic performance of the different cut-offs was comparable to that in the derivation cohort (Table 4). Yet, the 80% PCT decrease cut-off had appreciably lower sensitivity and negative predictive value regarding in-hospital mortality (79% and 77%) than it did in the derivation set, but almost identical specificity,. When PCT did not decrease or increased, the positive predictive value was high (52%) with high specificity (83%), similar to this criterion's performance in the derivation cohort.

## Discussion

This retrospective analysis of data from two independent US critical care settings found 72-hour PCT kinetics to be an accurate mortality predictor in intensively treated patients with sepsis. This finding was independent of initial severity assessment as reflected by the widely used APACHE IV or SAPS II clinical risk scores and validates similar results from Europe in patients with sepsis and severe systemic infections such as pneumonia [13-15]. Our data suggest that monitoring PCT kinetics in the first 72 hours of critical care provides information that may potentially help improve early transfer and therapy intensification decisions. Particularly, a PCT decrease >80% may help to identify individuals at reduced mortality risk who, therefore, are good early ICU discharge candidates. Conversely, a non-decrease or increase of PCT within this timeframe may help to flag patients who are at high mortality risk and, therefore, are likely to require treatment escalation.

Accurate disease severity assessment and clinical course prediction assist patients, families, and caregivers in setting realistic expectations regarding the illness. Accurate stratification and prognostication are also prerequisites for appropriately applying health care resources and therapeutic options. Informative communication with patients and significant others and effective patient management decision-making are particularly important in the care of severe stages of sepsis, which have a high morbidity and mortality risk. The value of prognostication is acknowledged by guidelines, which recommend stratifying patients with sepsis based on predicted mortality according to validated clinical risk scores, that is, SAPS or APACHE [18,19]. However, clinical risk scores are somewhat limited by practicality issues and are only validated when admission values are used; indeed, the utility of monitoring these scores over the course of sepsis is not well-established. The scores also may suffer from miscalibration, and, therefore, have only moderate operational characteristics, due to differences between the patient populations in whom the scores were developed and applied. Thus, there is interest in predictive use of newly available biomarkers that are objectively and rapidly measurable, respond to clinical recovery and add relevant, reliable, real-time information [20].

Although different clinical studies have produced results favoring PCT as a monitoring marker in sepsis and respiratory infections [13-15], there is currently a lack of widely-accepted cut-offs, that is, actionable biomarker levels, which would allow meaningful integration of PCT kinetics into patient prognostication in critical care. However, numerous randomized controlled trials have successfully employed PCT cut-offs for antibiotic stewardship in the medical and surgical ICU settings, mainly in patients with underlying respiratory infections, markedly decreasing antibiotic consumption without increasing mortality or treatment failure [11,14,21-23].

A smaller number of studies (reviewed in [14]) have evaluated the prognostic potential of PCT in sepsis, mainly looking at European patients. A Finnish investigation found PCT concentrations to be higher in more severe cases of advanced sepsis, but a substantial decrease in concentration was a more important survival predictor than were absolute values [13]. A 472-patient Danish study found a high maximum PCT level or a PCT increase for more than one day to be early independent predictors of 90-day all-cause mortality in ICU patients with sepsis [24]; these authors also observed that mortality risk rose with the number of days that PCT increased. Yet a large interventional trial testing the survival impact of therapy escalation in sepsis patients in whom PCT did not decrease appropriately could not show a benefit for PCT-guided escalation [12]; in fact, due to prolonged antibiotic therapy, patients in the intervention arm suffered more frequent complications including renal impairment and more ventilation days [25]. Although it remains unclear whether the PCT protocol could not identify the correct patients in whom therapy should have been escalated or whether the intervention was not beneficial *per se*, therapy escalation based on PCT concentrations cannot yet be recommended in the sepsis setting [26]. Importantly, the above-mentioned interventional study used daily PCT measurement to guide decisions concerning antibiotic therapy escalation. Our data suggest that a longer measurement interval, that is, 72 hours, may be an effective alternative strategy to support decision-making.

Interestingly, the presence and speed of CRP decrease also have been found to correlate with prognosis in ICU patients with severe pneumonia [6]. Specifically, the ratio of follow-up CRP measurements to the baseline measurement during the first week of therapy correlated closely with the individual clinical evolution. It will be interesting to compare the prognostic accuracy of PCT and CRP kinetics in a head-to-head study.

Importantly, there were differences in our two study populations. The derivation cohort had mostly white patients with confirmed severe sepsis or septic shock with higher initial PCT levels and a higher ICU mortality rate. The validation cohort had a lower overall severity with patients with a broader spectrum of sepsis or severe systemic infection and a lower ICU mortality rate. Moreover, the derivation cohort had a substantially greater proportion of women, and, as a result, an appreciably higher prevalence of the urinary tract as the primary locus of sepsis, while the validation cohort was much more diverse with respect to the primary locus of sepsis. These discrepancies may partly explain the differences in performance of 72-hour PCT kinetics, particularly when looking at the 80% decrease cut-off and in-hospital mortality. Future studies should address whether algorithms need to be adjusted in lower-severity patients, as is the case with antibiotic stewardship algorithms when applied in different clinical settings [21]. Such adjustment also may be needed when choosing the optimal negative and positive predictive values of PCT, which should depend on the outcome prevalence, and thus the expected risk, of the typical patient in the setting of interest.

Limitations of this work should be noted. As an observational retrospective analysis with patient inclusion based on medical records and with patients treated at different types of ICUs, the study may have had a selection bias. The results, therefore, need additional, prospective validation in a larger patient population. Our sample was too small to adjust for multiple confounders or to investigate effect modification by underlying illness or type of ICU. We restricted our analysis to patients with confirmed or likely sepsis who survived at least the first 60 hours of critical care, limiting the generalizability to other populations. Additionally, we used a different severity of illness score (APACHE IV or SAPS II) for adjusting the regression models in each cohort, reflecting routine institutional choice; use of different scores may limit comparability between our two cohorts. Also, the cohorts included different patient populations regarding their sepsis syndrome, which again limits comparability and calls for prospective validation in a well-defined patient cohort. Importantly, in a second step, interventional trials need to be conducted to ultimately characterize the impact of PCT monitoring on patient care and pharmacoeconomics.

## Conclusions

This analysis found that PCT kinetics over the first 72 hours of critical care provided prognostic information about ICU mortality and in-hospital mortality in patients with confirmed or likely sepsis independent of state-of-the-art initial clinical severity scores in two US settings, thereby extending observations of previous European studies. Further investigation is needed to validate these findings more definitively and to show whether monitoring PCT will improve patient outcomes and health care resource use.

## Key messages

• Close monitoring and repeated risk assessment of sepsis patients in the ICU is important for decisions regarding care intensification or early discharge to the ward.

• In this two-center, retrospective derivation-validation study, the change in PCT levels over the first 72 critical care hours in ICU patients with confirmed or likely sepsis showed a strong association with adverse outcome, including in-hospital and ICU mortality.

• The prognostic information derived from PCT kinetics was independent of initial clinical risk score (APACHE IV or SAPS II).

• A 72-hour PCT decrease >80% had a negative predictive value of around 90%; conversely, no decrease or an increase in PCT over 72 hours had a positive predictive value around 50%.

## Abbreviations

APACHE: Acute Physiology, Age and Chronic Health Evaluation; AUC: area under the curve; CI: confidence interval; CRP: C-reactive protein; LOS: length-of-stay; OR: odds ratio; PCT: procalcitonin; ROC: receiver operating characteristic.

## Competing interests

Thermo Fisher Scientific (formerly B·R·A·H·M·S AG) provided an unrestricted research grant to cover expenses for data acquisition and analysis. This firm had no involvement in design or conduct of this study, including the collection, management, analysis, or interpretation of the data, the decision to submit a report, or the preparation or approval of the manuscript.

PS, DA and EG received support from Thermo and bioMérieux to attend meetings and to fulfill speaking engagements. PS also has received additional research grants from Thermo. All other authors declare they have no competing interests.

## Authors' contributions

PS, DA and EG had the idea for, and initiated the study; VP, PM and AD organized the data collection; PS performed the statistical analyses and developed the initial draft of the manuscript. All authors amended and commented on the manuscript, revising it critically for important intellectual content. All authors read and approved the final manuscript.

PS, DA and EG are guarantors of the paper, taking responsibility for the integrity of the work as a whole, from inception to published article.
